# Cerebrospinal Fluid Biomarkers for Dementia with Lewy Bodies

**DOI:** 10.4061/2010/536538

**Published:** 2010-10-17

**Authors:** Elizabeta B. Mukaetova-Ladinska, Rachael Monteith, Elaine K. Perry

**Affiliations:** Institute for Ageing and Health, Campus for Ageing and Vitality, Newcastle University, Westgate Road, Newcastle upon Tyne, Newcastle NE5 5PL, UK

## Abstract

More than 750,000 of the UK population suffer from some form of cognitive impairment and dementia. Of these, 5–20% will have Dementia with Lewy Bodies (DLB). Clinico-pathological studies have shown that it is the low frequency of DLB clinical core features that makes the DLB diagnosis hardly recognisable during life, and easily misdiagnosed for other forms of dementia. This has an impact on the treatment and long-term care of the affected subjects. Having a biochemical test, based on quantification of a specific DLB biomarker within Cerebrospinal Fluid (CSF) could be an effective diagnostic method to improve the differential diagnosis. Although some of the investigated DLB CSF biomarkers are well within the clinical criteria for sensitivity and specificity (>90%), they all seem to be confounded by the contradictory data for each of the major groups of biomarkers (*α*-synuclein, tau and amyloid proteins). However, a combination of CSF measures appear to emerge, that may well be able to differentiate DLB from other dementias: *α*-synuclein reduction in early DLB, a correlation between CSF *α*-synuclein and A*β*42 measures (characteristic for DLB only), and t-tau and p-tau181 profile (differentiating AD from DLB).

## 1. Introduction

Presently, in the United Kingdom (UK), it is estimated that over 100,000 people have been diagnosed with Dementia with Lewy Bodies (DLB), accounting for 15–20% of the total number of recorded cases of dementia. By 2050, up to 1.8 million UK inhabitants will be affected by dementia [1], thus raising the number of DLB sufferers by 3-fold. These figures inevitably reflect the rapid increase in the ageing populations in UK and Europe. Thus, according to the National Office of Statistics (2009), by 2033, 23% of people in UK will be aged 65 or over, and of them 15–25% will have some form of cognitive impairment. 

### 1.1. DLB Clinical Symptomatology

Clinically, DLB is characterised by fluctuation of cognitive abilities alongside distinctive psychopathological symptoms, including recurrent, regular visual hallucinations and delusions [2]. Neurologically, 25–50% of the DLB patients have extrapyramidal symptomatology, including rigidity and bradykinesia, alongside hypophonic speech, masked facies, stooped posture, and a slow and shuffling gait, whereas the resting tremor is less common [3, 4].

A diagnosis of DLB requires presence of at least two core features (Table 1): fluctuating cognition; definite, regular hallucinations; or spontaneous parkinsonian movement disorders (present in 78% of DLB patients [4, 5]). In addition, suggestive features of the probable DLB diagnosis include Rapid Eye Movement (REM) sleep behaviour disorder, severe neuroleptic sensitivity, and low dopamine transporter uptake in basal ganglia as demonstrated by SPECT and/or PET imaging [5]. In studies using postmortem diagnosis of DLB as the gold standard, an appropriate diagnosis of DLB can be made with a sensitivity of 78–83% and a specificity of 85–95% (using [3] clinical criteria, [6, 7]), with the presence of visual hallucinations early in the course of dementia being a strong predictor of DLB at autopsy [8]. However, these values are achieved when the criteria are used in the specialist clinic, and thus should be considered as a maximal rather than an average value. Indeed the sensitivity and specificity for DLB diagnosis appear to be somewhat lower when related to subjects coming from hospital environment, with sensitivity and specificity being 60% and 85%, respectively [9].

### 1.2. Differentiation of DLB from Other Dementias

In the clinical setting DLB is commonly misdiagnosed as Alzheimer's Disease (AD) or Parkinson's Disease (PD) due to their overlapping clinical symptoms [10]. Differential diagnosis is essential, as around 50% of DLB patients are hypersensitive to conventional antipsychotic medication have worsening delusions and exacerbated motor symptoms [11].

The diagnosis of DLB, probable or possible dementia of AD, and vascular dementia (VaD) is largely based on clinical and neuropsychological assessment, using the current diagnostic criteria for different dementias (DSM-IV-TR and specific criteria for each disorder, e.g., NINCDS/ADRDA for AD, NINDS/AIREN for VaD, and International Consensus Criteria for DLB, not included in DSM-IV-TR criteria). The validity and reliability of these existing criteria have relatively good specificity, but low sensitivity for detecting distinct types of the dementing process, making them of limited value for routine clinical practice.

The neuroradiological investigations also have limitations in differentiating distinct types of dementia since many dementia sufferers have a degree of generalised brain atrophy, ventricular dilatation, white matter lesions, and/or ischaemic (sub)cortical changes, though a recent study showed that hippocampal atrophy [12] may differentiate DLB from AD and VaD. HMPAO SPECT occipital lobe hypoperfusion, largely thought to be characteristic for DLB [13, 14], has now been reported to be similarly present to an extent in other forms of dementia (see [15], reviewed in [16]). The latest studies measuring brain amyloid load by [^11^C]PIB-PET also show that not all individuals (89%) with a probable diagnosis of AD will have an increased amyloid brain load [17]. Similarly, many DLB subjects have widespread PIB binding [18], very similar to that seen in AD subjects. Recent studies have also demonstrated the usefulness of ^123^I-metaiodobenzylguanidine (MIBG) myocardial scintigraphy for the diagnosis of DLB, with marked reduction of cardiac MIBG uptake being a specific marker of Lewy body diseases [19, 20]. However, this procedure is not well validated as the DaTSCAN-SPECT and remains an unlicensed product for this indication. We have to highlight that a majority of the DLB MIBG myocardial scintigraphy studies (with exception of few, e.g., [21, 22]) have been conducted in Asia, where heart disease rates are low. Since MIBG myocardial scintigraphy can be abnormal in heart failure and, to some extent, severe ischaemic heart disease [23], further studies are needed to explore its clinical utilisation for DLB diagnosis in the Western countries where the prevalence rates of heart diseases are much higher (reviewed in [24]). 

### 1.3. Clinico-Neuropathological Correlates of DLB

The similarities between different dementias may be explained by the degree of overlapping pathology [25] and their attributable risks for cognitive impairment in the elderly [26]. In particular, coexisting AD pathology (tangles and plaques) is known to modify clinical symptoms, disease course, and progression. Thus, DLB individuals with additional neocortical tangles often lack the typical DLB symptom profile (e.g., lack of core symptoms including fluctuation, visual hallucinations, and parkinsonism), showing pronounced memory deficits, severe construction deficit, and a clinical presentation more characteristic for AD [27, 28]. Similarly, approximately 70% of DLB patients have neuropathological changes characteristic of AD, and, clinically, they tend to have more profound cognitive impairment than those with “pure” DLB [29]. Correspondingly, at least 59% of AD patients have LBs, usually restricted to amygdala and sparing the neocortical regions, with their number increasing as the disease progresses [30]. Interestingly, the presence of LBs in the amygdala appears to increase the risk for major depression in AD by nearly 5-fold [31]. Furthermore, in AD subjects with similar severity of cognitive impairment at baseline and comparable Braak stages at autopsy, those with concomitant neocortical LB pathology (referred to as Lewy body variant of AD) generally have faster cognitive decline and accelerated mortality compared to those with “pure” AD [32].

The neuropathological phenotype, as discussed above, influences the clinical presentation in dementia subjects. To date, the detection of the dementia hallmarks, for example, tangles and plaques (for AD), Lewy bodies and Lewy neurites (for DLB), and cerebrovascular changes (for VaD), with the exception of the amyloid deposits, that can be visualised with PIB) is largely confined to neuropathological assessment. The ultimate diagnostic goal, therefore, remains to develop methodology for peripheral detection of the molecular substrates of the characteristic dementia neuropathological hallmarks in the cerebrospinal fluid (CSF), blood and blood derivates and/or urine, to aid the diagnosis, and to monitor the treatment and progression of the disease process. This is particularly important in DLB, in the light of the difficulties of diagnosing this type of dementia in routine clinical setting.

### 1.4. Diagnostic Tools for DLB

The currently available neuroimaging techniques and associated diagnostic tests for dementia (also used to aid the diagnosis of DLB) represent a high financial burden to Health Care Systems (Table 2). These costs will inevitably rise on an annual basis in the light of the rapidly growing population, as reviewed above. In contrast, tests conducted using samples of bodily fluids (blood, urine, and CSF) are considerably less expensive, more readily available, and patient friendly.

Currently, there are no conclusive methods to test for DLB, and recommended CSF or other peripheral biomarkers for routine use in the differential diagnosis of DLB are lacking. Disease biomarkers, defined as “analytes in biological samples, (that provide) any measurement that predicts a person's disease state or response to a drug…” [33] need to fulfil additional criteria, for example, to reflect the central brain pathological process, be reproducible and have over a 90% specificity and sensitivity to changes in phenotype of the condition in order to allow for it to be considered clinically useful for diagnostic purposes [34]. From a clinical perspective, a single biomarker offering such diagnostic and prognostic capability would be the preferable choice.

The CSF contains an abundance of proteins, including neuron- and astrocyte-related and synapse-specific proteins [35]. Among these, the main constituents of Lewy bodies (*α*-synuclein), neurofibrillary pathology (tau protein), and amyloid deposits (A*β*) are detected. Their peripheral CSF detection provides an opportunity to aid the neurochemical diagnosis of various dementia processes. This is particularly important for DLB in the light of the diagnostic difficulties in routine clinical setting, as discussed above.

## 2. Materials and Methods

This paper is based on literature searches of several databases, including Medline/Ovid, Scopus, Web of Knowledge, and PubMed. All searches were restricted to literature published in English between the years 1980–2010 (January 1980–June 2010). For the purpose of this paper we concentrated on research studies only on CSF biomarkers and did not include biomarker studies conducted in bloods and/or blood derivates (including plasma/serum), urine, or genomic studies. Review articles were also included if they addressed either individual CSF biomarkers or in the context of CSF proteomic studies conducted in DLB and Lewy body disease. In addition, all articles were reviewed for possible relevant references. The key words used for these searches were Dementia with Lewy bodies (DLB), Lewy body, cerebrospinal fluid (CSF), biomarkers, proteomics, amyloid protein, tau protein, and synuclein.

### 2.1. CSF Biomarkers for DLB

#### 2.1.1. *α*-Synuclein as a DLB Biomarker

***α*-Synuclein Processing and Role in DLB**

The main components of LBs are amyloid-like fibrils composed of *α*-synuclein (also referred to as non-amyloid component of plaques or NACP, synelfin, and SYN1). Although the actual function of *α*-synuclein remains ambiguous, the protein is involved in both synaptic rearrangements and, as a chaperone in the formation of SNARE (Soluble NSF Attachment protein receptors) complexes, is implicated in the synaptic vesicle trafficking in the human nervous system (reviewed in [36]).

*α*-Synuclein is a relatively small (112–140 amino acids in length) presynaptic nonsecreting protein and makes up around 1% of the total protein within the brain and less than 0.001% of the CSF proteome [37]. The *α*-synuclein phosphorylation, occurring on serine 129, is crucial in mediating *α*-synuclein neurotoxicity and in modulating protein structure, including fibrillar aggregation (see [38], reviewed in [36]). The hippocampal CA2 region is thought to be especially vulnerable to this *α*-synuclein post-translational modification [39]. Similarly, mutations in the *SNCA*, or Synuclein-Alpha (non-A4 component of amyloid precursor) gene [40] as well as the overproduction of *α*-synuclein [41] in some rare cases of familial DLB give rise to the characteristic pathology in DLB, and provide further insights into the altered processing of this synaptic protein.

**Detection of CSF *α*-Synuclein in DLB**

The monomeric, soluble *α*-synuclein (molecular weight 14–19 kDa), but not the higher molecular species (e.g., oligomers and polymers, present in blood and brain tissue), is found in the CSF see [42, 43]). However, the presence of higher molecular aggregates (>150 kDa) consisting of *α*-synuclein, complement C3 precursor, complement C4-B precursor, and Ig gamma-1 chain -C region in the absence of soluble monomeric *α*-synuclein, has also been described [44].

The limited number of currently available studies has provided rather conflicting results regarding the measures of CSF *α*-synuclein in DLB subjects: decreased and unchanged to even elevated levels have all been reported (see Table 3, [45]). Furthermore, a recent study found that, although *α*-synuclein detection in CSF may not be useful for differentiating DLB from AD, the CSF *α*-synuclein measures were significantly correlated with duration of the DLB (*P *< .05), but not with the AD [46]. This would suggest that the reduction of the CSF *α*-synuclein may reflect the extent of Lewy-related pathology characteristic of more advanced stages of DLB [47]. It must be noted, though, that the Noguchi-Shinohara et al. study [46] did not include control subjects, and as an alternative, a group of “matched” DLB patients with similar MMSE score results to the AD patients were included, instead.

*α*-Synuclein CSF levels appear to be similar in various dementia types, including DLB, AD, frontotemporal dementia (FTD) or VaD, and those of control subjects [48]. However, the findings for AD subjects were not conclusive, and lower levels of CSF *α*-synuclein, compared to controls, have also been reported (*P *< .001; Table 3; see [49]). In the latter study, the decrease in *α*-synuclein in the CSF of AD subjects was significantly associated with the disease duration, suggesting that it well reflects the extent of advanced AD neuropathology and profound brain synaptic loss, including *α*-synuclein as described previously [50].

In contrast to the above studies, Mollenhauer et al. [51] and Kasuga et al. [52] reported differences in CSF *α*-synuclein levels between AD, PD, DLB, and control subjects. There was a significant decrease in CSF *α*-synuclein in PD and DLB individuals, in comparison to the AD and control groups (*P*  =  .025; see [51]) and those with other dementias, including FTD, PSP, VaD, normal pressure hydrocephalus, and unspecified dementias (*P* < .01; see [52]). The *α*-synuclein gene (SNCA) duplication, which in some instances is associated with somewhat more aggressive clinical presentation of both motor and cognitive symptoms [53], appears not to influence the *α*-synuclein expression in the CSF, since the affected carriers with the DLB/PDD clinical phenotype have a similar CSF *α*-synuclein levels as DLB individuals [52].

The *α*-synuclein levels appear not to be associated with the extent of cognitive impairment (as assessed by MMSE), DLB, or AD disease duration, age, or gender [52]. However, the *α*-synuclein CSF levels are correlated with those of A*β*42 and restricted to DLB subjects [52], suggesting that there may well be a close relationship between the amyloid and *α*-synuclein brain processing and deposits in DLB subjects. In a recent study, the significant reduction in the *α*-synuclein CSF levels was present even in DLB subjects with mild dementia [44], further indicating that lower CSF *α*-synuclein protein may be an early marker for the disease.

**Methodological Limitations of Available *α*-Synuclein Analytical Tools**

The reasons as to why various studies are markedly different must be addressed in order to promote better study designs. Thus, it has been questioned whether the technique recommended by Tokuda et al. [83] (which requires concentration of CSF samples) could lead to inaccuracies. In addition, the assays used in some of the studies introduced incubation of the CSF samples for 48 hours at 4°C. The latter experimental step is associated with oligomerization of the *α*-synuclein *in vitro*, and thus may be a contributing factor for both the observed decrease and variability in the reported concentration of *α*-synuclein in CSF.

The choice of immunoprobes (N-terminal end being more consistently present in the CSF than the C-terminal end portion of the protein), as well as differential expression of distinct *α*-synuclein isoforms (with a modified C-terminus) in ageing and neurodegenerative disorders, may also underlie the reported differences in the detection of *α*-synuclein in the CSF (reviewed in [36, 45]). Similarly, the findings of decrease in *α*-synuclein in DLB as a function of the disease duration [46] may reflect the central neuropathological process of the disease and the consequent molecular changes associated with the latter. The duration-dependent decrease in CSF *α*-synuclein suggests the extent of *α*-synuclein aggregates in DLB [84] and their inability to pass the brain blood barrier [84, 85]. We have also previously reported a significant depletion of *α*-synuclein in more advanced stages of AD, preceded by a transient upregulation of the protein in both CSF and brain tissue occurring in the Braak stage 4 [45, 50, 86]. Further correlative clinico-neuropathological studies will need to follow to explore the neuropathological correlates of the *α*-synuclein CSF changes in DLB and associated dementia syndromes.

#### 2.1.2. Tau Protein as a DLB Biomarker

**Tau Protein Detection in DLB**

**Figure 1:**
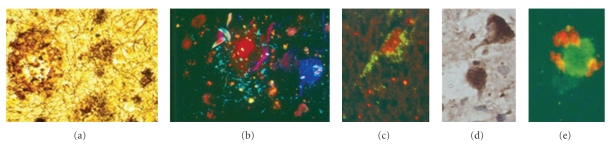
Neuropathological hallmarks of dementia. Senile plaques (a) containing amyloid protein and neurofibrillary pathology (b) consisting of altered tau protein are the hallmarks of Alzheimer's disease and ageing. In dementia, loss of synaptic proteins affects both allo- and neocortical areas. In Dementia with Lewy body, intraneuronal aggregates of *α*-synuclein give rise to Lewy bodies ((d),(e)) that are also found in normal ageing and in other neurodegenerative diseases, including AD and PDD. Light microscopy ((a), (d)); confocal microscopy ((b), (c), (e)). Labelling: Bielschowsky silver (a), tau immunohistochemistry (b), synaptophysin labelling (c), and alpha-synuclein immunohistochemistry ((d), (e)). Magnification: x100 (a) and x400 ((b)–(e)).

The microtubule-associated Tau Protein represents an integral component of the paired helical filaments (PHFs). The truncation of the protein at Glu391 and/or its phosphorylation (regulated by a number of kinases) are the crucial step in the self-assembly of the protein into PHFs, found within various neurofibrillary structures (e.g., neurofibrillary tangles, neuritic plaques, and dystrophic neurites; see Figure 1) commonly associated with many neurodegenerative disorders, such as AD and DLB [87].

Tau proteins are also present in the CSF and have been extensively investigated in various dementia syndromes. However, this protein (which is also referred to as Beta-2 transferrin or desialyated transferrin) is not routinely found in blood or other body fluids, and its presence has been detected in plasma only transiently, following a stroke [88].

**Phosphorylated Tau Protein in DLB CSF**

Since hyperphosphorylated tau protein occurs in neurodegenerative disorders associated with neurofibrillary pathology, it is not surprising that phosphorylated tau epitopes, for example, threonine 231 (p-tau231), threonine 181 (p-tau181), and serine 199 (p-tau199), have been recommended by a consensus group as promising biomarkers to differentiate AD from other dementias, provided that they have a sensitivity level of 85% or greater and a specificity level of at least 75% [89]. These posttranslational modifications of the tau protein have been extensively assessed in various types of dementias, and in particular how well they differentiate DLB from AD using CSF samples [57]. These studies have indicated that p-tau231 is the initial post-translational modification of the tau protein found in the CSF, potentially making it a key biomarker in the early detection of tauopathies, for example, AD [90].

**CSF p-tau181 in DLB**

Although p-tau231 is a relatively consistent biomarker of all dementias (including DLB), increased concentrations of p-tau181 in the CSF appear to be often implicated in DLB (see [91], Table 3). The CSF elevation of p-tau181 is not restricted to DLB, but also found in several other neurodegenerative disorders, thus reflecting the presence of overlapping neurofibrillary pathology. Nevertheless, p-tau181 measures appear to be different in the two dementia types: AD subjects have significantly higher CSF level of p-tau181 compared to control and DLB subjects (see [59]; Table 3), and this differentiates AD from DLB, with a sensitivity of 91% and a specificity of 94% [60]. However, in autopsy-confirmed AD, the diagnostic accuracy of CSF p-tau181 to discriminate AD from DLB showed lower sensitivity and specificity (75% and 61%, respectively, and 73% diagnostic accuracy; see [61]).

The data from the latter study were similar to those of Hampel et al. [57] which found an increase in p-tau181 CSF measures to have a sensitivity of 94% and a specificity of 64% for differentiating AD and DLB. However, the combination of p-tau231 and p-tau199 did not produce promising results in differentiating between these two dementia syndromes: specificities of p-tau231 and p-tau199 were 64% and 50–64%, respectively. The latter may be due to the low sensitivity and specificity of p-tau199 CSF measures (both ranging between 25–30%) in differentiating between AD, other dementia subtypes, and control subjects [60].

**CSF Total Tau and Relationship to p-tau Measures in DLB**

One of the earliest attempts in evaluating the potential of CSF measures of total tau protein (t-tau) in dementia studies was that by Arai et al. [54], which reported significantly elevated CSF t-tau protein levels in AD subjects in comparison to PD patients (Table 3). In the follow-up study [92], CSF t-tau levels were determined in a number of dementia syndromes including FTD, progressive supranuclear palsy (PSP), corticobasal degeneration (CBD), and DLB, as well as a control group. Although CSF t-tau was elevated in the DLB group, there was no significant difference between the DLB and AD subjects (using the previous data from the AD patients). In contrast, Parnetti et al. [55] reported differences in both CSF t-tau and p-tau levels between AD and DLB patients with a greater difference for the p-tau CSF (Table 3).

These findings of higher t-tau and p-tau CSF measures in AD subjects in comparison to other forms of dementia were confirmed again in a later study by the same group (see [58]; Table 3), with the DLB subjects, although having lower t-tau than AD (*P  * =  .039), still exhibiting 2-3 fold higher level of CSF t-tau measures than those in PD, PDD, and control subjects. Furthermore, data from this study indicated that, of the 19 patients with DLB, half displayed high levels of t-tau in their CSF, similar to those of the AD subjects. Interestingly, PD subjects with dementia also showed an elevation of t-tau and p-tau compared with PD and control groups, and this was also accompanied by a decrease in amyloid peptides [76], similar to previous dementia studies. Similar findings of highly elevated CSF t-tau and p-tau181 have now been reported for some autopsy-confirmed DLB patients [93].

Concentrations of both t-tau and p-tau do not correlate with the DLB disease duration [58]. However, significant inverse correlation between t-tau levels and MMSE (*r* = −0.54; *P * = .02) along with a Milan Overall Dementia Assessment (MODA) (a standardised assessment for staging dementia used globally but developed within Italian clinics [94]) (*r* = −0.66; *P* = .002) score has been reported, similar to findings of a previous study [55]. One of the explanations for this may be the significantly lower levels of t-tau and p-tau181 already present in incipient DLB [62], suggesting that the cognitive changes may well be influenced by additional factors, for example, neuronal cell loss, vascular insults, and so forth.

**CSF Tau Protein Changes in Autopsy-Proven DLB**

The presence of LBs may have a damaging effect upon the neuronal cytoskeleton (reviewed in [36]), and thus, may contribute to the altered levels of tau within the CSF in DLB subjects. Indeed, elevated levels of CSF total-tau (considered a marker of axonal neuronal damage) have been confirmed in cases with a definite diagnosis of AD, Lewy Body variant of AD, as well as DLB alone (see [56]; Table 3), thus reflecting the described impairment in axonal transport [95] and axonal loss (reviewed in [96]) underlying the development of both AD and LB pathologies. However, the CSF t-tau findings are not conclusive, and a contrary report of a decrease in t-tau in DLB was also described in a similar study conducted on autopsy-confirmed sample [61]. The latter may well reflect the more advanced stages of the dementia disease process, characterised by both generalised axonal and neuronal loss, as reported previously (see [97], reviewed in [98]).

Recent correlative biochemical and neuropathological studies have also highlighted the relationship between the CSF tau measures (p-tau181 and p-tau231) with the extent of brain neurofibrillary pathology (e.g., neuritic plaques and neocortical neurofibrillary tangles) in AD subjects [99, 100], thus confirming that the CSF tau protein measures reflect closely the brain accumulation of the characteristic AD hallmarks of the disease, the neurofibrillary pathology. However, the findings for CSF p-tau181 are not conclusive, as previous studies have reported lack of association of this CSF tau measurement with neurofibrillary pathology [101, 102]. The differences in the reports may arise from the differences in timing of obtaining the CSF samples in relation to autopsy (ranging from approximately one year [101, 102] to 6 years [99]), suggesting that the CSF p-tau measures close to death do not necessarily reflect the true extent of neurofibrillary pathology in the brain, as detected using immunohistochemical [101] or immunobiochemical [90] methods, since the presence of end stages of neurofibrillary tangles (the so-called “ghost tangles”, consisting of the core of the paired helical filaments; see [103]) could not have been addressed.

#### 2.1.3. Amyloid-Beta (A*β*) Peptides as a Biomarker

**A*β* Processing**

A*β* peptides play an important role not only in the AD pathogenesis [104], but also in DLB. It is suggested by interacting with *α*-synuclein that the amyloid peptides promote aggregation, enhance the accumulation of *α*-synuclein pathologies, and accelerate cognitive dysfunction [105]. It is, therefore, reasonable to examine amyloid peptides for their potential diagnostic value in DLB.

**CSF A*β* Peptides in DLB**

The altered brain processing of APP, leading to accumulation of extracellular amyloid deposits throughout the brain tissue of the affected individuals, is also seen in the periphery, for example, CSF and blood/blood derivates. Thus, the CSF decrease of A*β* peptide 1-42, although characteristic of AD, is also found in DLB and PDD (see [64]; Table 3), and this may reflect the similar extent of A*β* deposits in these diseases (reviewed in [106]). In support of the latter are the recent findings of CSF A*β*42 loss in DLB subjects being accompanied by lower CSF levels of cystatin C [107], a peptide that inhibits fibril formation and oligomerization of the amyloid peptide [108]. Further evidence for the role of the amyloid brain deposits in downregulating CSF A*β*42 levels comes from a PET imaging study, which demonstrated that the PIB binding in DLB was associated with cognitive impairment (*P* = .0006) and decrease in CSF A*β*42 (*P* = .042; see [18]).

The shorter amyloid peptide (A*β*1-40) also appears to be decreased in the CSF of AD subjects, and significantly more in those with a clinical diagnosis of DLB and vascular dementia [48]. Furthermore, the ratio between the longer and shorter CSF amyloid peptides (A*β*42/A*β*40) appears to be superior in discriminating between AD and other neurodegenerative disorders, including DLB, than the A*β*42 measure alone (*P*<  .01), with the former being equally robust as the combination of A*β*42, p-tau181, and t-tau (see [63]; Table 3). In contrast, in AD (but not DLB and PDD) there was a slight increase in CSF A*β*1-37 [64], and this may reflect the slight differences in the A*β* brain deposition between AD and DLB, in terms of the deposition occurring later in the course of DLB [109] or the faster rate of disease progression in DLB [32].

In the Bibl et al. [64] study, a novel A*β*-like peptide (considered to be an oxidised *α*-helical form of A*β*, designated as A*β*1-40*) was detected in all recruited participants (including the nondemented controls) and was significantly increased in those affected by DLB (in comparison with PDD patients), and to a lesser degree in AD as compared to PDD and control subjects. In fact, the CSF A*β*1-40* measures had 81% sensitivity and 71% specificity at differentiating between DLB and PDD. Furthermore, the ratio of A*β*1-42 to A*β*1-37 significantly differentiated the control subjects from those with DLB, PDD, and AD and differentiated the AD subjects from those with DLB and PDD [64]. These findings suggest that the A*β* CSF patterns vary between AD, DLB, and PDD, and introduction of A*β* ratios improves the diagnostic CSF test accuracy for the dementia differential diagnosis, which is not the case when sole measurements of A*β*1-42 are reported. Similarly, the differential diagnostic value of A*β* peptide patterns in combination with tau protein assays appears to be improved. The ratio of A*β*1-42 to A*β*1-38 and A*β*1-42 to A*β*1-37, when combined with t-tau levels, has 100% sensitivity and 92% specificity in differentiating AD from DLB and control subjects [65].

A study by Parnetti et al. [58] investigated a wider range of subjects, including those with DLB, PD, PDD, and AD. In comparison to PD, PDD, and AD subjects, the DLB group had the lowest CSF level of A*β*42, and the latter was negatively correlated with the dementia duration. In addition, DLB patients had a significantly higher t-tau CSF measures relative to PD, PDD, and controls. However, differences in CSF levels of p-tau, although significantly elevated in AD, failed to discriminate the disease entities within the Lewy body disease (LBD) spectrum, irrespective of presence of dementia (DLB, PDD, or PD). It is important to note that 4 out of 23 AD patients in this study had a CSF analytes composition remarkably similar to that of the DLB group.

The above set of data is similar to that of Vanderstichele et al. [110]. The latter group reported a significant decrease in CSF A*β*42 in both AD (*P* < .001) and LBD (*P* =  .002) patients, relative to the control group (data for DLB subjects were not extracted separately in this study). In contrast to their previous findings [64], later CSF studies, further supported by ^123^I-MIBG cardiac scintigraphy observations, found no significant difference in the CSF A*β*42 measures between AD and DLB subjects [65, 67]. However, the cardiac scintigraphy provided the best discrimination between the two dementia groups. Thus, the DLB subjects had significant elevation of washout rate in comparison to both the AD and control groups [67].

### 2.2. Miscellaneous Biomarkers

**Inflammatory Markers**

The biochemical and neuropathological studies in DLB have highlighted a number of novel putative molecular candidates present in the CSF (Table 3). Inflammation is associated with amyloid accumulation in dementia [111], and inflammatory markers have also been detected and investigated in the CSF. However, in DLB, the interleukin CSF levels, specifically, IL-1*β* and IL-6, did not discriminate DLB from control subjects [112]. Similarly, CSF measures of the precursor peptides for enkephalins and substance P (midregional proenkephalin A and N-terminal protachykinin A, resp.), involved in inflammation and pain, although decreased in dementia disorders, including DLB, appear not to have the power to differentiate various dementia syndromes from acute neuroinflammatory disorders [113], suggesting that the CSF measures of these two neuropeptide precursor fragments could reflect the extent of neuroinflammation and reduction in neuronal activity, common among these diseases.

**CART Neuropeptides**

The hypothalamic region in DLB shows profound atrophy on MRI brain scans, and this may underlie the characteristic fluctuating clinical symptoms in this dementia syndrome. Thus, any alterations in molecular patterns associated with these characteristic morphological changes can be useful in developing a biomarker for DLB. The neuropeptide Cocaine and Amphetamine-Regulated Transcript (CART) is expressed selectively in neurons in the hypothalamic region. A study by Schultz et al. [71] reported a significant reduction by 30% in the CSF CART levels in DLB compared to both control and AD subjects. The depletion of CART may be a causative factor in the dysfunction of the dopaminergic system seen in DLB. Interestingly, CSF CART levels correlate with p-tau protein levels, but they do not appear to correlate with the DLB disease progression [71]. This suggests that neuroimaging techniques that could detect dysfunction of the dopaminergic system (a consistent finding in various DLB studies [114]) could also be a key in the future diagnosis of DLB.

**Brain Neurotransmitters**

Brain neurotransmitters play an essential role in various psychological and cognitive functions. The relatively widespread cholinergic [115, 116] and dopaminergic [117, 118] changes in DLB indicate a more generalised neurotransmitter dysfunction that may also be detected in the periphery. To test this hypothesis, Molina et al. [70] measured the concentration of various amino acids (AA) considered to reflect the neurotransmitter changes within the CSF. Of these, levels of asparagine and glycine, but not glutamate, glutamine, aspartate, and GABA, were raised in both CSF and plasma from DLB patients compared with age-matched controls, with only the plasma levels reaching statistical significance. This suggests that the plasma measures of asparagine and glycine may be useful for the DLB diagnosis. Interestingly, higher levels of glycine are also found in other neurological disorders.

**Posttranslational Protein Modifications**

*In vitro* oxidation and nitration of *α*-synuclein is associated with the aggregation of this protein [119], which leads to the characteristic intraneuronal filamentous inclusions characteristic of DLB pathology. Interestingly, a study conducted by Molina et al. [69] found an increase in Nitric Oxide (NO), associated with protein nitration processes, in CSF in DLB patients in comparison to the control group. Not only does this give an indication of further pathological processes within DLB, but it also highlights the need for further research to determine the clinical relevance of NO as a biomarker for the diagnosis of DLB.

**Markers of Cytoskeletal Changes**

An increase in neurofilament (NF) in the CSF is also indicative of neuronal degeneration, as seen in neurodegenerative disorders, especially AD. However, De Jong et al. [91] did not find any NF elevation within CSF of DLB patients relative to patients affected by late onset AD, despite an increase in cortical NF containing neurons in DLB. This indicates that peripheral NF concentration does not reflect the cortical NF expression. One of the limitations of this study was the small sample size and the lack of postmortem verification of the diagnosis. Further studies on larger clinical samples are now needed to overcome these limitations.

**Metal Homeostasis**

Dysfunction in metal homeostasis has been implicated as a causative factor for neurodegeneration. In a study conducted by Bostrom et al. [68] measurements of magnesium (Mg), calcium (Ca), iron (Fe), copper (Cu), zinc (Zn), rubidium (Rb), strontium (Sr), and caesium (Cs) were taken from the CSF samples. Ca and Mg levels were elevated in DLB compared to AD, VaD, and control subjects. In this study, the combined Ca and Mg CSF measurements had a sensitivity of 93% and a specificity of 85% (using cutoff values of ≤48.0 mg/L for Ca and ≤27.3 mg/L for Mg) to differentiate DLB from AD. Since clinical criteria state that biomarkers must have a specificity and a sensitivity of ≥80% (as discussed above [120]), the latter findings may well support the use of Ca and Mg CSF measures as potential biomarkers for DLB.

**Thyroid Hormone-Binding Protein Transporter of Thyroxine**

Transthyretin (TTR) changes are not only characteristic for familial amyloid polyneuropathy, but also for other neurodegenerative disorders, such as AD and dementia in general [121]. It is thought that TTR has a “neuroprotective” role in AD, via the prevention of formation of A*β* fibrils. Despite TTR proteins having a strong linkage with DLB-type pathology, the study by Schultz et al. [72] did not find any correlation between CSF concentrations of TTR and clinical presentation of DLB.

The clinical designs of the above studies (sample size, inclusion criteria, differences in clinical assessments, number of dementia types analysed, defining control groups, etc.) all differed, and these differences need to be considered when interpreting the results. With the exception of the CSF measures of CART and metal compounds, there are no putative biomarkers that emerge to differentiate DLB from normal ageing and/or other types of dementia.

### 2.3. Additional CSF Biomarkers in Parkinson's Disease (PD)

The overlapping clinical symptoms between DLB and PD make the differentiation of these two clinical entities difficult. Not surprisingly, our searches for DLB CSF biomarkers highlighted a number of studies that included PD and PDD subjects. While these studies may not ultimately highlight a suitable biomarker for DLB, they should be considered, even if only for evidence of further exclusion criteria.

**BDNF**

Brain-derived neurotrophic factor (BDNF) is important for sustaining existing neurons while promoting the growth of new neurons. Therefore, depletion of BDNF could account for the loss and damage of neurons during various neurodegenerative disorders. In PD, elevated CSF concentrations of BDNF relative to those of control subjects have been reported [78]. Since BDNF is a significant mediator of PD pathology, its role should be assessed further in other neurodegenerative conditions, specifically DLB due to similarities with PD in terms of existing pathology.

**Dopamine and Dopamine Metabolites**

As discussed above with respect to assessment of the transmitter system, indirect measurement of Dopamine (DA) concentration via its metabolites, Homovanillic acid (HVA), and Dihydroxyphenylacetic acid (DOPAC) may give additional information about factors that contribute to the pathology of neurodegenerative conditions where DA dysfunction occurs. One particular study measured DA, HVA, and DOPAC in different stages of PD [77]. This study also reported an exponential decrease of total DA in the CSF with disease progression, with a rapid drop during the initial phases of the disease onset, whereas an increased HVA/DA ratio (which indicates DA turnover) correlated largely with disease duration.

**Hypocretin-1**

Another potential biomarker highlighted due to the recognition of sleep-related disorders in neurodegenerative conditions is hypocretin -1. This peptide was initially associated with regulation of the sleep/wake cycle, along with various autonomic dysfunctions. It has since been identified in the CSF. A recent study conducted on PD subjects found a 40% decrease of hypocretin-1 in the prefrontal cortex and a 25% decrease in the ventricular CSF when compared to controls [122]. Using CSF samples from a wider range of neurological disorders, for example, DLB, progressive supranuclear palsy (PSP), corticobasal degeneration (CBD), along with PD, can help to characterise the pathological substrates of these sleep-related disorders [123]. However, while sleep disorders are symptomatically linked with these dementia syndromes, it seems as though one of the major indicators of sleep dysfunction (the reduction of orexin) is not altered in the CSF [74, 123].

**Metals**

Metals can contribute to both prooxidant and antioxidant processes within the body. Investigations of the substantia nigra in PD patients have demonstrated an increase of iron (Fe), which, due to the production of free radicals during its catalysis, could be a major contributor to oxidative damage [124]. This makes it the metal of interest when pursuing possible biomarkers for PD, along with Chromium (Cr) and Lead (Pb), which have been linked to PD cases. These three metals have been shown to be significantly reduced in CSF of PD patients relative to those of the controls [73]. However, these observations have to be evaluated in comparison to DLB patients.

## 3. Conclusions

Despite the neuroradiological advances aiding the clinical diagnosis of distinct subtypes of dementias, their overlapping clinical and neuropathological features make clear differentiation difficult in clinical practice. Similarly, the dementia clinical symptomatology varies from patient to patient, along with disease progression and severity. This has an impact on the pharmacological treatment and long-term care of the affected subjects. It is particularly important for DLB patients who have severe side effects to antipsychotic medication. 

Although a number of research studies provide evidence that some of the investigated CSF biomarkers are well within the clinical criteria for sensitivity and specificity (>90%), they all seem to be characterised by the contradictory data for each of the major groups of biomarkers: *α*-synuclein, tau, and amyloid proteins. Similarly, results from the miscellaneous biomarkers studies have proved disappointing and nonconclusive. Having said that, a combination of CSF measures appear to emerge, which may well be able to differentiate DLB from other dementias: *α*-synuclein reduction in early DLB, a correlation between CSF *α*-synuclein and A*β*42 measures (characteristic for DLB only), and t-tau and p-tau181 profile (differentiating AD from DLB). Their usefulness in clinical setting needs to be explored further.

Identifying highly specific and sensitive peripheral analytes that reflect the key hallmarks of dementia that can be used in clinical setting is an imperative. Such analytes have been successfully identified for AD, having high sensitivity and specificity in differentiating this neurodegenerative disorder from other forms of dementia. Further work concentrating on improving the currently available CSF *α*-synuclein analytical tools may lead to further insights about the peripheral *α*-synuclein processing, which, either alone, or in a combination with known or novel analytes, may aid the differential diagnosis of DLB. In this respect, CSF proteomic studies (reviewed in [125]) have provided promising results, for example, identifying eight novel proteins which can differentiate between AD, PD, and DLB with a 95% specificity and sensitivity (see [79]; Table 5). Similarly, proteomic studies based on specific pathological features of neurodegenerative conditions have also proved noteworthy. By exploiting the fact that substantia nigra (SN) is the most vulnerable region within the brain to be affected by oxidative stress, Basso et al. [80] collected postmortem tissue samples from this region from subjects with PD and reported that 9 of the 44 proteins had a changed pattern of expression in the PD patients compared with the controls. The most significant of these proteins was the upregulation of peroxiredoxin II, a characteristic indication of oxidative stress (Entrez Gene).

Although promising, further DLB proteomic studies are warranted for better methodological approaches to include a larger number of well- defined samples and be able to address either the central or associated brain disease (dementia) process(es) and how they are reflected in the periphery (CSF, blood/blood derivates, and/or urine). Their further testing in routine clinical settings, alongside with the currently available clinical screening and diagnostic tools, should enhance the early dementia diagnosis and also aid the monitoring and therapeutic outcomes for DLB-affected subjects.

## Figures and Tables

**Table 1:** Clinical diagnostic criteria for DLB.

Core features	Features supportive of diagnosis
A progressive, significant cognitive decline	Repeated falls
Fluctuations in cognition	Syncope
Recurrent, well-formed visual hallucinations	Transient loss of consciousness
Spontaneous motor features of parkinsonism	Neuroleptic sensitivity
	Systemized delusions
	Hallucinations in other modalities

Adapted from Mckeith et al. [5].

**Table 2:** Cost and time implications of neuroimaging and neurophysiological techniques and laboratory tests.

Neuroimaging technique	Cost (*£*)	Time (minutes)
CT	80–100	10
MRI	200	30
SPECT	250	15–60
DaTSCAN-SPECT	750	180–360
MIBG myocardial scintigraphy	500	180
EEG	250	30–45
Blood	2.71	5
Urine	2.12	5
CSF	10	35–40

Abbreviations: CT: computerised tomography; MRI: magnetic resonance imaging; SPECT: single-photon emission computed tomography; DaTSCAN-SPECT: [123I]ioflupane single-photon emission computed tomography; MIBG: ^123^I-metaiodobenzylguanidine; EEG: electroencephalogram; CSF: cerebrospinal fluid.

Please note that all costs and times are estimates based on UK NHS data and do not include professional interpretation of results.

**Table 3:** CSF biomarkers for DLB.

Study	Biomarker(s)	Technique	No. of subjects	No. of controls	Results
Mollenhauer et al. [51]	*α*-synuclein	Sandwich ELISA	38 DLB; 13 AD; 8 PD; 8 CJD	13 neurological controls	In PD and DLB, *α*-synuclein levels significantly reduced (*P* = .0305) compared to AD and control subjects.
Mukaetova-Ladinska et al. [45]	*α*-synuclein *γ*-synucleinIgG	Dot blot	5 LBD; 9 AD; 3 VaD	8	Postmortem ventricular CSF analysis. Elevation of both *α*- and *γ*-synucleins in AD, LBD, and VaD compared to controls. An increase in *α*- and *γ*-synucleins seen from Braak stage III onwards. Results not influenced by age at death or postmortem delay.
Noguchi-Shinohara et al. [46]	*α*-synuclein	ELISA Assay	16 DLB; 21 AD	(A subgroup of 13 DLB patients matched for duration of disease and MMSE score to those of the AD subjects)	*α*-synuclein levels do not differ between DLB and AD patients. Lower levels of *α*-synuclein in CSF correspond to DLB duration (*P* < .05).
Ohrfelt et al. [49]	*α*-synuclein	ELISA	15 DLB; 66 AD; 15 PD	55	Similar levels of *α*-synuclein in PD, DLB, and control subjects, whereas, in AD, *α*-synuclein levels significantly lower compared to controls (*P *< .001). AD subjects with MMSE <20 had significantly lower level of *α*-synuclein than AD subjects with MMSE ≥20.
Spies et al. [48]	*α*-synuclein	ELISA	40 DLB; 131 AD; 39 FTD; VaD 28	Two groups: Group A 57 (aged > 50); Group B 55 healthy volunteers	No significant difference in *α*-synuclein levels between DLB, AD, FTD, VaD, or control subjects.
Ballard et al. [44]	*α*-synuclein	Western blot	12 DLB	9	Significant lower levels of *α*-synuclein in DLB compared to controls (*P *< .05). Mildly cognitively impaired DLB subjects (MMSE > 24) also had lower levels than controls (*P *< .007).
Kasuga et al. [52]	*α*-synuclein/ t-tau/p-tau181/A*β*42	ELISA	34 DLB (including 2 with SNCA duplication); 31 AD; 21 other dementias (12 FTD; 2 PSP, 2 normal pressure hydrocephalus; 2 VaD; 3 unclassified)	No control group	*α*-synuclein significantly lower in DLB than in AD (*P* < .05) or other dementias (*P* < .01). CSF *α*-synuclein levels correlated with A*β*42 level in DLB only (*r* = 0.43; *P* = .01). CSF t-tau and p-tau181 levels as well as A*β*40/A*β*42 ratio levels significantly lower in DLB in relation to AD (*P *< .01), but similar to other dementias.
Arai et al. [54]	t-tau	Sandwich ELISA	6 DLB; 8 FTD; 6 PSP; 3 CBD	19 (data taken from previous study)	Similar levels of tau in AD and DLB (*P* = .78), but higher than in controls.
Parnett et al. [55]	t-tau/p-tau181/A*β*42	HT7-AT270 Assay and ROC analysis	43 DLB; 80 AD	40	Strong correlation between t-tau and p-tau independent of diagnostic group (*r* = 0.904). No differences between DLB and AD for A*β*42. The significant increase in p-tau181 in AD (*P *= .039) has 80% sensitivity at differentiating between AD and DLB.
Clark et al. [56]	t-tau/A*β*40/A*β*42 (includes also postmortem correlation)	ELISA	3 DLB; 74 AD (including 4 genetic AD and 10 LBVAD); 10 FTD; 5 CJD; 3 GSS syndrome; 11 miscellaneous neurologic conditions	73	DLB subjects had 2-fold higher level of t-tau in relation to controls, but two-fold lower levels in relation to AD. No differences in t-tau between LBVAD and AD (*P* = .30). A*β*42 highly depleted in DLB in comparison to both control (8-fold) and AD (4.4-fold) subjects.
Hampel et al. [57]	p-tau181/p-tau231/p-tau199	ELISA	22 DLB; 108 AD; 24 FTD; 7 VaD; 22 OND	23	Decrease in p-tau199, p-tau231, and p-tau181 (*P *< .001) in DLB compared to AD, with similar levels to other studied dementia groups.
Parnetti et al. [58]	t-tau/p-tau181/ Ab*β*42	ELISA	19 DLB; 23 AD; 20 PD; 8 PDD	20	DLB mean CSF t-tau levels significantly lower than in AD patients (*P* = .039), but significantly higher in PD, PDD, or control subjects. p-tau181 elevated in AD, but similar between DLB, PD, and PDD groups.
Vanderstichel et al. [59]	t-tau/ p-tau181/ A*β*42	ELISA assay	60 DLB; 94 AD	60**	Higher levels of p-tau181 in AD than in DLB and controls. p-tau181 was the most statistically significant single variable of the 3 biomarkers to discriminate between AD and DLB.
Simic et al. [60]	t-tau/ p-tau181/ p-tau199	ELISA assay	2 DLB; 11 AD; 5 FTD; 8 VaD	13	p-tau181 differentiates AD and DLB with a sensitivity of 91% and a specificity of 95%.
Koopman et al. [61] (autopsy confirmed dementias)	t-tau/p-tau181/A*β*42	ELISA	18 DLB; 95 AD; 10 FTD; 6 CJD; 16 VaD	No control group	DLB group had similar level of t-tau, p-tau181, and A*β*42 as the other dementia groups (FTD, CJD, and VaD); these dementia subjects had significantly lower t-tau (*P* = .025) and p-tau (*P *< .0001) and higher A*β*42 (*P* = .001) in comparison to AD.
Mattson et et al. [62] (autopsy confirmed dementias)	t-tau/p-tau181/A*β*42	ELISA	750 MCI (420 stable MCI; 14 incipient DLB; 271 incipient AD; 28 incipient VaD; 7 incipient FTD; 10 other dementias); 529 AD	304	MCI subjects who developed DLB had significantly lower levels of t-tau and p-tau181 at baseline compared to AD and incipient AD (*P* < .01), significantly lower A*β*42 CSF levels in relation to control (*P* < .001), stable MCI, and AD subjects (*P* < .01).
Spies et al. [63]	A*β*1-42/A*β*1-40/t-tau/p-tau181	ELISA	16 DLB; 69 AD; 26 VaD; 27 FTD	47	Significantly lower levels of A*β*40 in DLB and VaD in relation to AD (*P* < .01). AD had similar A*β*40 level to controls (*P* = .384). The A*β*42/A*β*40 ratio significantly lower in AD in comparison to other dementia groups (*P* < .001). A*β*42/A*β*40 ratio improves differentiating AD from VaD, DLB, and FTD than A*β*42 measures alone (*P *< .01). A*β*42/A*β*40 ratio performed equally well as the combination of A*β*42, p-tau181, and t-tau in differentiating AD from FTD and non-AD dementias.
Bibl et al. [64]	A*β* peptides	A*β*-SDS-PAGE/ immunoblot	21 DLB; 23 AD; 21 PDD	23	The significant increase of a novel peptide with an A*β*-like immunoreactivity (A*β*1-40*) in DLB patients relative to PDD has a sensitivity of 81% and a specificity of 71% using a cut of point of 0.954% but failed to be classified as a sole biomarker.
Bibl et al. [65]	A*β* peptides/tau	A*β*-SDS-PAGE/ immunoblot and ELISAs for A*β*1-42 and tau	25 probable DLB; 18 probable AD	14	The ratio of A*β*1-42/A*β*1-38 and A*β*1-42/A*β*1-37 when combined with absolute tau levels produced a diagnostic test with 100% sensitivity and 92% specificity. This ratio discriminated between AD and DLB with a high specificity (*P *= 6.6 × 10^−6^).
Wada-Isoe et al. [66, 67]	A*β*42/p-tau 181	ELISA assay	22 DLB; 34 AD	37	No significant difference in p-tau levels in AD and DLB, but a significant increase in the p-tau/A*β*42 ratio in AD in comparison to DLB.
Vanderstichele et al. [59]	A*β*42	ELISA	6 LBD; 39 AD; 10 other dementias, neurological and psychiatric disorder patients*	12	Significant decrease in CSF A*β*42 levels in both AD (*P *<.000l) and LBD (*P* = 0.002), relative to the control group.
Maetzler et al. [18]	A*β*42	ELISA	9 DLB; 12 PDD; 14 PD no dementia	No control group	Lower levels of A*β*42 in DLB and PDD compared to the nondemented PD subjects (*P *= .024). DLB-PIB-positive subjects had lower levels than the PIB-negative subjects (*P* = .044), but similar A*β*42 levels to the PIB-negative subjects who had dementia (*P *= .42).
Boström et al. [68]	Mg/Ca/Cu	Mass spectrometry	29 DLB	51	Levels of Mg/Ca/Cu increased in CSF in DLB relative to controls, although increases in Cu not significant. The CSF-Mg concentration had a sensitivity of 93% and a specificity of 81% to detect DLB.
Molina et al. [69]	Nitric-oxide metabolites (L-arginine to L-citrulline)	Ionic -exchange chromatography	22 DLB	13	Not statistically significant difference in NO metabolite concentration between DLB and controls.
Molina et al. [70]	Neurotransmitter (NT) amino-acid (AA) concentrations	Ion-exchange chromatography	21 DLB	26**	No significant differences between control and DLB groups in relation to glutamate, aspartate, and GABA levels; however, higher concentrations of asparagine (+25%) and glycine (+21%) in DLB.
Schultz et al. [71]	Cocaine- and Amphetamine-Regulated Transcript (CART)	Radio-immunoassay	12 DLB; 14 AD	12	Significant decrease (30%) in CART in DLB versus controls (*P* < .0001), DLB, and AD (*P* < .05), but concentrations of CART did not indicate DLB progression. CART levels correlated with p-tau protein concentration.
Schultz et al. [72]	Transthyretin (TTR)	Radio-immunoassay	13 DLB; 59 AD	13	No significant differences of TTR concentrations between AD and DLB.

Please note that ELISA assays for t-tau, p-tau, and A*β*42, unless otherwise specified, refer to commercially available sandwich ELISA assays.

Abbreviations: DLB: Dementia with Lewy bodies; LBD: Lewy Body disease; AD: Alzheimer's Disease; LBVAD: Lewy Body variant of Alzheimer's disease; PD: Parkinson's Disease; PDD: Parkinson disease dementia; CJD: Creutzfeldt-Jakob disease; GSS: Gerstmann-Straussler-Scheinker syndrome; FTD: Frontotemporal dementia; VaD: Vascular Dementia; PSP: Progressive supranuclear palsy; OND: other neurologic disorders (e.g., mild psychiatric or neurologic symptoms); CSF: Cerebrospinal fluid; A*β*: Amyloid-beta peptide; t-tau: total tau; p-tau: phosphorylated tau; ELISA: Enzyme-Linked Immunosorbent assay; A*β*-SDS-PAGE: A*β*-sodium dodecylsulphate-polyacrylamide gel electrophoresis; ROC: Receiver Operating Characteristic; MMSE: Mini Mental State Examination; PIB: ^11^C-labelled amyloid ligand Pittsburgh Compound B.

*Specifically vascular dementia (*n* = 3); hypoxia during cardiac arrest (*n* = 1); cerebrovascular lesion (*n* = 1); unspecified dementia (*n* = 2); depression (*n* = 3).

**Age-matched control.

**Table 4:** CSF studies in Parkinson's disease.

Study	Type(s) of Biomarker	Sample(s) taken	Type(s) dementia
Almonti et al. [73]	Metals	CSF	PD
Asai et al. [74]	Orexin	CSF	PD
Bibl et al. [75]	Amyloid-Beta/ Tau	CSF/Plasma	VaD
Compta et al. [76]	Amyloid-Beta/ Tau	CSF	PD/PDD
Lunardi et al. [77]	DA and metabolites*	CSF	PD
Salehi and Mashayekhi et al. [78]	BDNF	CSF	PD

BDNF: Brain-Derived Neurotrophic Factor; DA: Dopamine; CSF: Cerebrospinal fluid; VaD: Vascular Dementia; PD: Parkinson's Disease; *Homovanillic acid (HVA), Dihydroxyphenylacetic acid (DOPAC).

**Table 5:** Proteomic studies.

Study	Technique(s) used	Sample(s) taken	Type(s) of dementia
Abdi et al. [79]	iTRAQ	CSF	DLB/AD/PD
Basso et al. [80]	MALDI-TOF-MS	SN tissue	PD
Davidsson et al. [81]	2D gel electrophoresis	CSF	AD
Wada-Isoe et al. [66]	SELDI-TOF-MS	Serum	DLB/AD
Yin et al. [82]	LC-MS/MS and 2-DE	CSF	AD/PD
